# Ultrasound-guided pericapsular nerve group (PENG) block for early analgesia in elderly patients with hip fractures: a single-center prospective randomized controlled study

**DOI:** 10.1186/s12871-023-02336-1

**Published:** 2023-11-23

**Authors:** Yan Tang, Xinlei Zhang, Shuai Yi, Dan Li, Hui Guo, Yunqing Liu, Jindong Liu, Mingjian Kong

**Affiliations:** 1 Department of Anesthesiology, The Second Affiliated Hospital of Xuzhou Medical University, No. 32, Meijian Road, Xuzhou, Jiangsu Province China; 2 Department of Anesthesiology, The Affiliated Hospital of Xuzhou Medical University, No. 99 Huai Hai West Road, Xuzhou, Jiangsu Province China

**Keywords:** PENG block, Flurbiprofen, Hip fractures, Elderly patients, Early analgesia

## Abstract

**Background:**

The aim of this study was to compare the efficacy of ultrasound-guided PENG (pericapsular nerve group) block and drug therapy with intravenous flurbiprofen for early analgesia in elderly patients with hip fractures after hospitalization.

**Methods:**

This is a single-center, observer-blinded, prospective, randomized, controlled trial. A total of 41 elderly patients (aged 60 or older) with hip fractures were enrolled in the current study. Patients were randomly assigned to two groups: Group P (ultrasound-guided PENG block, 20 mL of 0.375% ropivacaine) and Group F (intravenous flurbiprofen 50 mg). The primary outcome measure was the dynamic (passive straight leg raising 15°) NRS (numerical rating scale 0 to 10) pain scores at different time points. The secondary outcomes were the static NRS scores at different time points, the number of rescue analgesia sessions, patient satisfaction, and the incidence of complications.

**Results:**

Patients in the two groups had comparable baseline characteristics. The group P had lower dynamic and static NRS scores at 15 min, 30 min, 6 h, and 12 h after intervention (*P*<0.05) than the group F. The highest NRS pain scores in the group P were still lower than the NRS scores in the group F at 30 min-12 h (Group F: 5.57±1.54 vs. Group P: 3.00±1.12, *P*<0.001), and there was no significant difference between the two groups at 12-24 h (Group F: 6.35±1.79 vs. Group P: 5.90±1.83, *P*>0.05). The group P had higher satisfaction scores (Group P: 9 (9,9) vs. Group F: 8 (7,8), *P*<0.001). There was no statistically significant difference in the number of rescue analgesics at 0-12 h or 12-24 h or the incidence of complications between the groups.

**Conclusions:**

Compared with intravenous flurbiprofen, ultrasound-guided PENG block provides better early analgesic effects in elderly patients with hip fractures, and a PENG block is safe for elderly patients with hip fractures after hospitalization.

Trial registration

This study was registered in the Chinese Clinical Trial Testing Center (ID: ChiCTR2200062400).

**Supplementary Information:**

The online version contains supplementary material available at 10.1186/s12871-023-02336-1.

## Introduction

Approximately 1.5 million people experience hip fractures each year, and as the population grows and increases in age [1], the number of elderly patients with hip fractures is estimated to increase to 2.6-7.3 million by 2025 and to 4.5-21.3 million by 2050 [2]. Pain after hip fracture has been associated with confusion, depression, sleep disturbance and poor recovery [3, 4]. Mismanagement of pain has been associated with delayed walking, pulmonary complications, delayed hospitalization and cardiovascular complications [3, 5, 6]. The most significant pain associated with a hip fracture is dynamic pain, which will inevitably occur during clinical examination and nursing care after admission. Therefore, patients with hip fractures should undergo early pain assessment to receive the best analgesic treatment as soon as possible. This will help manage acute pain caused by hip fractures [7].

It has been demonstrated that nonsteroidal anti-inflammatory drugs (NSAIDs) can relieve pain and are better for functional improvement [8, 9]. Low to standard doses of NSAIDs may be considered in the short-term treatment of acute pain related to fractures, fracture repair, or other acute musculoskeletal injuries when the bleeding risk is not substantial [9].

Flurbiprofen is NSAIDs that has potent anti-inflammatory, analgesic, and antipyretic activities and is used globally [10]. One of their primary mechanisms of action is the inhibition of COX (cyclo-oxygenase) to exert an analgesic effect [10–12]. Flurbiprofen 50 mg intravenously was fully hydrolyzed to flurbiprofen within 5 min, which reached the highest blood concentration (8.9 ug/mL), and it has the characteristics of quick starting effect. In our hospital, flurbiprofen is commonly prescribed before orthopedic surgery.

In 2018, Girón-Arango et al described the PENG (Pericapsular Nerve Group) block, which can be applied to hip fracture and has the ability to reduce the median dynamic pain score by 7 points in hip fracture patients [13]. Short, Kitcharanant et al [14, 15] confirmed through an anatomical study that PENG block targets the articular branches of the femoral nerve, obturator nerve and accessory obturator nerve [13]. Furthermore, some studies have demonstrated that a PENG block provides better pain relief and is easier to apply during spinal anesthesia [7, 16, 17]. To date, there are few clinical trials that have analysed the early analgesia of this technique in elderly patients with hip fractures after hospitalization. We, therefore, conducted an observer-blinded, prospective, randomized, controlled trial to evaluate the efficacy of the PENG block in improving early analgesia in elderly patients with hip fractures after hospitalization. The primary outcome measure was the dynamic (passive straight leg raising 15°) NRS (numerical rating scale 0 to 10) pain scores at different time points. The secondary outcomes were the static NRS scores at different time points, the number of rescue analgesia sessions, patient satisfaction (using a scale of 0-10, 10 being the most satisfied), and the incidence of complications.

## Methods

### Study design and subjects

The study conformed to the guidelines of the Declaration of Helsinki and was approved by the Ethics Committee of The Second Affiliated Hospital of Xuzhou Medical University ([2022] 052501), and this study was registered with the Chinese Clinical Trial Testing Center (ID: ChiCTR2200062400,04.08.2022). Written informed consent was obtained from all the subjects participating in the trial. The study plan was to enroll patients beginning in June, but patients were actually recruited between August 2022 and December 2022 after clinical registration. Inclusion criteria: ① Patients with an imaging diagnosis of hip fracture; ② Age ≥60 years old; ③ Dynamic NRS scores ≥4 points; ④ BMI: 18-30 kg/m^2^. Exclusion criteria: ① Patients with allergies to the drug used in this experiment; ② Patients with local or systemic infection; ③ Patients with coagulopathy; ④ Patients with severe cardiopulmonary insufficiency; ⑤ Patients with a history of ipsilateral hip surgery; ⑥ Patients with mental, language, communication, or hearing impairment; ⑦ Patients who refused to participate in this trial; ⑧ Patients with multiple systemic injuries.

### Randomization, blinding and study intervention

A nurse not involved in the study used Excel to generate a random integer set of 1-41, odd number into group F and even number into group P. These random numbers were placed in sealed opaque envelopes, and the anesthesiologist experienced in performing pericapsular nerve group block conducted the trial intervention. Group P received ultrasound-guided PENG block with 20 mL of 0.375% ropivacaine) and Group F received intravenous flurbiprofen 50 mg. In this study, a resident anesthesiologist who was unaware of the randomization assignment collected the data. Another member of this group who was blinded to the other steps of this test performed statistical analysis and remained blinded throughout the entire process.

**Fig. 1:**
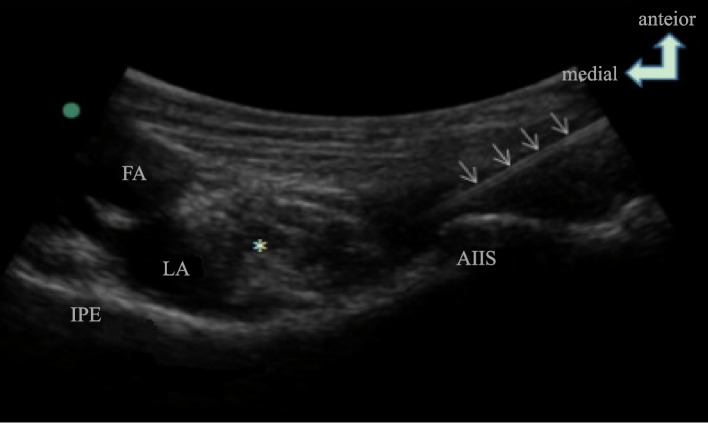
Representative image and ultrasound scan of patient receiving PENG block. Notes: White arrow is ultrasound-guide tracing of puncture needle; * Iliopsoas tendon is marked. Abbreviations: PENG, pericapsular nerve group; AIIS, anterior inferior iliac spine; IPE, iliopubic eminence; FA, femoral artery; LA, local anesthetics

Patients in the group P received the PENG block as described by Girón-Arango [11]. The regional block was performed with the patient in the supine position. A curvilinear low-frequency ultrasound probe (2-6 MHz, Sonosite) was initially placed in a transverse plane over the anterior inferior iliac spine and then aligned with the pubic ramus by rotating the probe counterclockwise approximately 45 degrees. The iliopsoas eminence, the iliopsoas muscle and tendon, the femoral artery, and the pectineus muscle were observed. A 22G puncture needle was inserted from lateral to medial in an in-plane approach to place the tip in the musculofascial plane between the psoas tendon anteriorly and the pubic ramus posteriorly. Following negative aspiration, 2 mL of normal saline was injected to identify the correct location of the tip, followed by an injection of 20 mL of 0.375% ropivacaine (Naropin, AstraZeneca, eg, Fig. 1). Patients in the group F received intravenous 50 mg of flurbiprofen.

After those with contraindications were excluded, both groups received analgesia (Ultrasound-guided PENG block or intravenous flurbiprofen) as soon as possible after admission. When the NRS scores≥ 4 at rest after analgesia, the patients recevied intramuscular 5 mg dezocine as rescue analgesia. Dezocine was used repeatedly, if required, with dosing intervals of no less than 6 hours.

### Outcome measures

The basic information of all the patients was collected, including the static and dynamic NRS scores after the intervention. In addition, we collected pulse oxygen saturation (SpO_2_), mean arterial pressure (MAP), and heart rate (HR). The primary outcome was dynamic NRS scores (passive straight leg raising 15°) at 15 min, 30 min, 6 h, 12 h, and 24 h after the intervention. Secondary outcomes were static NRS score at 15 min, 30 min, 6 h, 12 h, and 24 h after the intervention, the highest NRS scores and number of rescue analgesics at 0-12 h and 12-24 h, the incidences of nausea, vomiting, bleeding at the puncture site, hematoma, and local anesthetic poisoning reaction within 24 h. Within 24 h after the intervention, overall satisfaction was scored on the VAS in which scores range from 0 to 10, with 0 indicating ‘not satisfied’ and 10 indicating ‘most satisfied’. If the patient slept between 23:00 and 7:00, we thought the pain was mild at this time, so the mean static NRS score was 1.5 points, thus indicating mild pain and the maximum dynamic NRS score was 3 points, also indicating mild pain.

NRS scores at 30 min is 30 min after the intervention, with the NRS score at this moment unrelated to the other interventions, and this same is also true for NRS scores at 6 h, 12 h, 24 h.

### Statistical analysis

The sample size was determined to be sufficient through calculations using the GPower 3.1.1 computer program software. The power analysis indicated that a total of 34 participants, including a 15% dropout rate (number of measurements = 6), were needed for a medium partial η^2^ (0.25) when α = 0.05 for a power of 0.95 with 2 independent groups, using a repeated measures analysis of variance (ANOVA) and within-between subject interactions. A total of 38 participants, including a 15% dropout rate, were needed when the time before the intervention was not considered (number of measurements = 5).

The obtained data were analyzed with IBM SPSS Statistics 25 (IBM, Armonk, NY, USA). The parametric distribution of numerical variables was evaluated by the Shapiro–Wilk normality test. The Levene test was used to verify homogeneity. The normally distributed measures are denoted as the mean ± standard deviation (mean ±SD). The T test was applied to make independent comparisons between groups. Repeated measures ANOVA was used to analyze the repeated variables. Nonnormally distributed measures were expressed as the median and interquartile range (IQR) or marginal means from generalized estimation equations and standard deviation from raw data among persons with information at specific time points (mean ±SD)^a^. Mann‒Whitney U test or Generalized Estimation Equations for nonparametric continuous variables. In one patient with missing scores at 24 h, we did not interpolate. Moreover, the chi-square test or Fischer’s exact test was applied to compare categorical variables. A two-tailed *P* <0.05 was considered statistically significant.

## Results

**Fig. 2:**
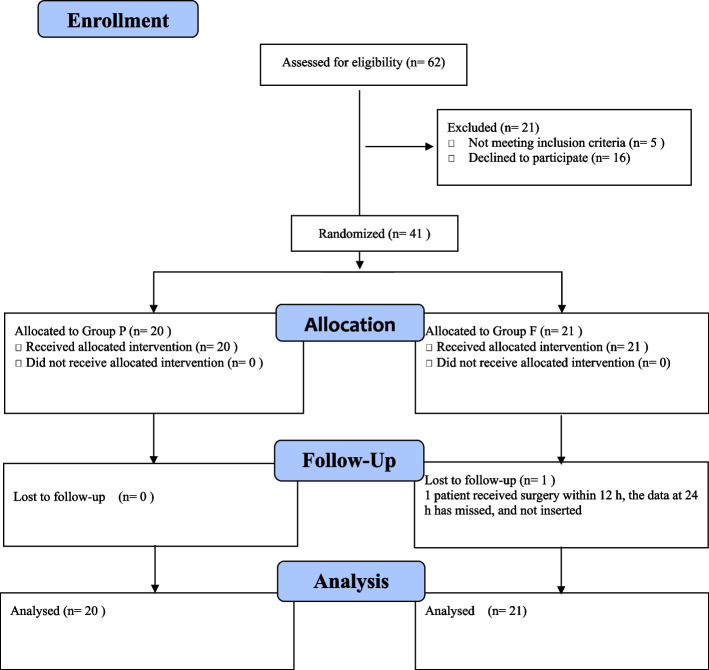
Flow diagram of the study

Of the 62 patients who were assessed for eligibility, 16 patients refused to sign informed consent, 3 patients had hearing impairment, and 2 patients had a history of hip surgery on the affected side and were therefore not included. The remaining 41 patients were randomly and equally allocated between the groups. One patient in the group F underwent surgery within 12-24 h, so there were missing data at 24 h, and the remaining data of this patient were entered into the statistical analysis (eg, Fig. 2). Subject characteristics are presented in Table 1, and no clinically relevant differences were apparent from the group characteristics.

**Table 1:** Subject characteristics by study group

Variable	Group F (*n*=21)	Group P (*n*=20)	*P*
Age (y); (mean ± SD)	78.67±5.92	76.45±4.56	0.188
BMI (kg.m^-2^); median (IQR)	20.2 (19.1-22.8)	22.16 (20.1-23.6)	0.262
Female/ Male (n/n)	18/3	16/4	0.697
Left/Right side fracture (n/n)	11/10	12/8	0.623
Transcervical fracture/ Intertrochanter fracture of femur (n/n)	17/4	15/5	0.719
ASA physical status; I/ II/ III (n/n/n)	2/5/14	3/7/10	0.595
Basic disease			
Coronary disease; n (%)	7 (33)	6 (30)	0.819
Cerebral infarction;n (%)	18 (85.7)	14 (70)	0.179
Diabetes; n (%)	4 (19)	2 (10)	0.663
Hypertension; n (%)	8 (38.1)	12 (60)	0.161
Respiratory disease; n (%)	3 (14.3)	1 (5)	0.606
Other diseases; n (%)	10 (47.6)	12 (60)	0.427
MAP before intervention; (mean ± SD)	81.43±7.79	85.7±9.71	0.127
HR before intervention; (mean ± SD)	82.10±5.63	81.95±5.94	0.936
SpO_2_ before intervention; median (IQR)	98 (96-98)	98 (97-98)	0.140
Dynamic NRS score before intervention; median (IQR)	9 (8-10)	9 (6-10)	0.146
Static NRS score before intervention; median (IQR)	4 (4-5)	4.5 (3-5)	0.463

Data are presented as mean ± SD, median ( IQR), number (%) or number.

Abbreviations: *SD* Standard deviation, *IQR* Interquartile range, *BMI* Body mass index, *ASA* American Society of Anesthesiologists, *MAP* Mean arterial pressure, *HR* Heart rate, *SpO*_*2*_, pulse oxygen saturation, *NRS* Numerical rating scale. *P*<0.05 was considered statistically significant

### Primary outcome

The dynamic NRS scores were analyzed using generalized estimation equations, which showed interaction effects for time and group (*P*<0.001), and the dynamic NRS score was further analyzed. The dynamic NRS scores were significantly different between the two groups at 15 min (95% CI 2.437-4.420, *P*<0.001), 30 min (95% CI 2.75-4.65, *P*<0.001), 6 h (95% CI 1.89-3.41, *P*<0.001), and 12 h (95% CI 1.05-2.42, *P*<0.001), and the dynamic NRS scores of the group P were lower than those of the group F. There was no significant difference in dynamic NRS scores between the two groups at 24 h (95% CI -0.92-0.72, *P*=0.810) (eg, Table 2).

**Table 2:** Dynamic NRS scores at different time points

Variable	Time	Group F	Group P	95% CI	*P*
Dynamic NRS scores (mean ± SD)^a^	before intervention	8.76±1.48	7.95±1.99	0.54 (-0.24-1.86)	0.129
	15 min after intervention	6.43±1.96	3.00±1.38	3.43 (2.44-4.42)	<0.001
	30 min after intervention	6.00±1.93	2.30±1.22	3.70 (2.75-4.65)	<0.001
	6 h after intervention	4.95±1.28	2.30±1.22	2.65 (1.89-3.41)	<0.001
	12 h after intervention	4.29±1,26	2.55±1.05	1.74 (1.05-2.42)	<0.001
	24 h after intervention^**^	5.05±1.15	5.15±1.53	-0.10 (-0.92-0.72)	0.810

Abbreviations: *NRS* Numerical rating scale, *SD* Standard deviation

^a^Marginal means from generalized estimation equations and SDs from raw data among persons with information at specific time points

^**^The sample size of group F is 20.* P*<0.05 was considered statistically significant

### Secondary outcomes

**Fig. 3:**
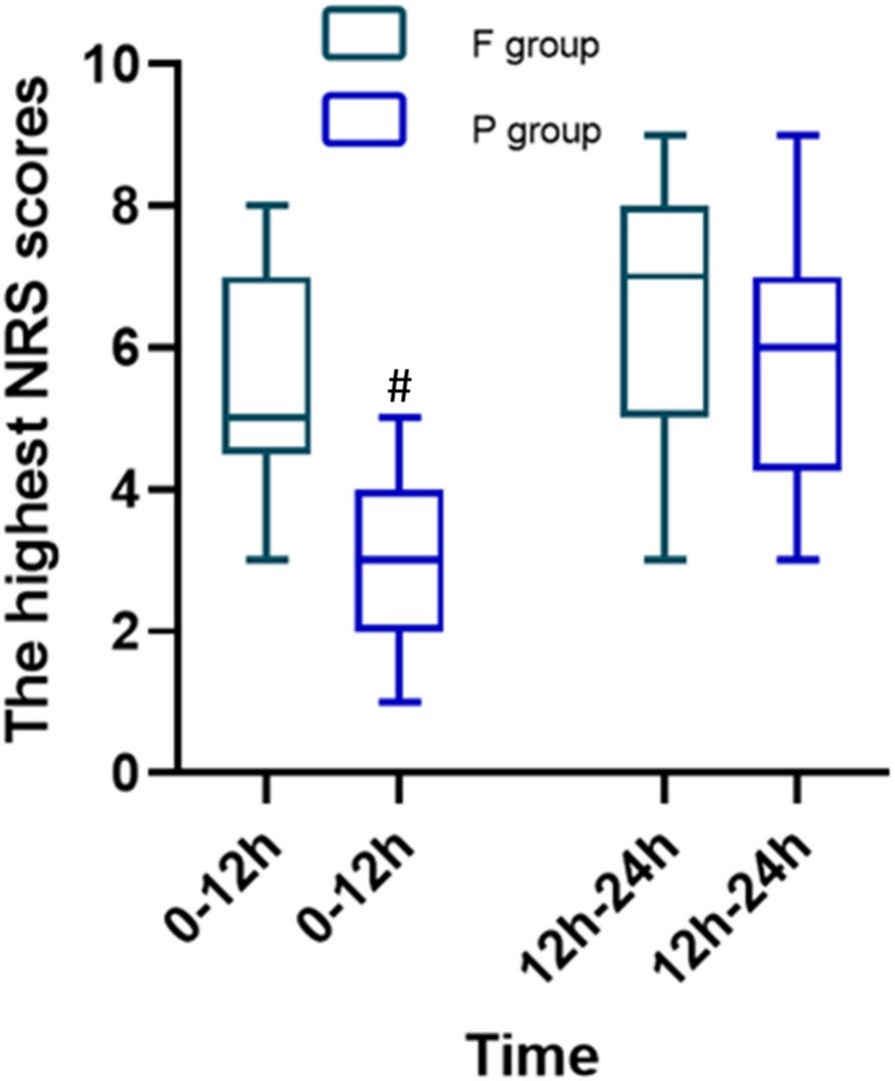
The highest NRS scores. Notes: ^#^*P* < 0.05, there was significant difference between the two groups

The static NRS scores were analyzed using generalized estimation equations, which showed interaction effects for time and group (*P*<0.001), and the static NRS scores were further analyzed. The static NRS scores were significantly different between the two groups at 15 min after intervention (95% CI 0.71-2.01, *P*<0.001), 30 min after intervention (95% CI 1.07-2.39, *P*<0.001), 6 h after intervention (95% CI 0.56-1.65, *P*<0.001), and 12 h after intervention (95% CI 0.97-2.26, *P*<0.001), and the static NRS scores of the group P were lower than those of the group F. There was no significant difference in the static NRS scores between the two groups at 24 h after intervention (95% CI -027-0.86, *P*=0.220) (eg, Table 3). Although the highest NRS scores at 0-12 h were lower in the group P than in the group F and the difference was statistically significant between the two groups (Group F: 5.57±1.54 vs. Group P: 3.00±1.12, *P*<0.001) (Table 4), there was no difference in the highest NRS scores at 12-24 h (Group F: 6.35±1.79 vs. Group P: 5.90±1.83, *P*=0.436) (eg, Fig. 3). The incidence of rescue analgesia was lower in the group P than in the group F at 0-12 h (Group F: 38% vs. Group P: 10%) and 12 h-24 h (Group F: 65% vs. Group P: 40%), but there was no statistically significant difference in the incidence of rescue analgesics administered at 0-12 h and 12 h-24 h (*P*>0.05). Overall satisfaction was higher in the group P within 24 h (*P*<0.001). The incidence of nausea and vomiting was similar in the two groups, with no significant difference (*P*>0.05). There was no local hematoma or infection in the group P.

**Table 3:** Static NRS scores at different time points

Variable	Time	Group F	Group P	95% CI	*P*
Static NRS scores (mean ± SD)^a^	before intervention	4.52±0.99	4.15±1.14	0.37 (-0.26-1.01)	0.249
	15 min after intervention	2.81±1.42	1.45±0.69	1.36 (0.71-2.01)	<0.001
	30 min after intervention	2.48±1.43	0.75±0.72	1.73 (1.07-2.39)	<0.001
	6 h after intervention	1.90±1.05	0.80±0.77	1.11 (0.56-1.65)	<0.001
	12 h after intervention	2.74±1.36	1.13±0.74	1.61 (0.97-2.26)	<0.001
	24 h after intervention^**^	2.75±1.02	2.40±0.82	0.29 (-027-0.86)	0.220

Abbreviations: *NRS* Numerical rating scale, *SD* Standard deviation, *IQR* Interquartile range

^a^Marginal means from generalized estimation equations and SDs from raw data among persons with information at specific time points

^**^The sample size of group F is 20.* P*<0.05 was considered statistically significant

**Table 4:** Other secondary outcome measures

Variable	Time	Group F	Group P	*P*
The highest NRS scores; (mean ± SD)	0-12 h	5.57±1.54	3.00±1.12	<0.001
	12 h-24 h	6.35±1.79^**^	5.90±1.83	0.436
Rescue analgesia; n (%)	0-12 h	8 (38)	2 (10)	0.067
	12 h-24 h	13 (65) ^**^	8 (40)	0.113
Overall satisfaction; median (IQR)	0-24 h	8 (7-8) ^**^	9 (9-9)	<0.001
Nausea; n (%)	0-24 h	3 (15) ^**^	2 (10)	1
Vomit; n (%)	0-24 h	0 (0) ^**^	0 (0)	1

Data are presented as mean ± SD, median (IQR) or number (%)

Abbreviations: NRS, numerical rating scale; SD, standard deviation; IQR, interquartile range

^**^The sample size of group F is 20.* P*<0.05 was considered statistically significant

In addition, we also performed an intragroup comparison and found that both groups had reduced static and dynamic NRS scores (eg, Supplementary Tables, Table S1). We also analyzed MAP, HR and SpO_2._ Statistical analysis of MAP, HR and SpO_2_ showed no interaction effect between MAP and HR (*P*>0.05) and no statistically significant difference between time and group (*P*>0.05) (eg, Supplementary Tables, Table S2). Both groups had no interaction effect on SpO_2_ (*P*>0.05) but were significantly different over time. The SpO_2_ at 15 min and 30 min after intervention was higher than before intervention (*P*<0.05), which we believe may be related to oxygen inhalation after admission (eg, Supplementary Tables, Table S3).

## Discussion

In this study, PENG block was compared with intravenous flurbiprofen in the management of early analgesia in elderly patients with hip fracture. The results showed that both PENG block and intravenous flurbiprofen resulted in effective pain control, but when compared with intravenous flurbiprofen, PENG block provided better analgesic effects and higher satisfaction with analgesia.

PENG block is a new and promising ultrasound-guided regional anesthesia technique that aims to block the branches of the femoral nerve, obturator nerve and accessory obturator nerve innervating the anterior hip capsule [13, 18, 19]. In previous studies, a PENG block has been proven to be effective in reducing hip-related pain [16, 19–21].

In this prospective, randomized study of 41 elderly patients with hip fractures, one patient had sequelae of cerebral infarction; and although the muscle strength of the left limb was decreased and that of the right limb (fracture side) was normal, we do not think this affected our trial. The PENG block provided analgesia to elderly patients with hip fractures, thus reducing the dynamic and static NRS scores at 15 min, 30 min, 6 h, and 12 h after intervention and the highest NRS scores at 0-12 h compared to intravenous flurbiprofen. Our findings are similar to previously published reports by Pascarella et al [19]. This study showed that the PENG block didn’t reduce the incidence of salvage analgesia compared with intravenous flurbiprofen. This finding was not consistent with the study by Pascarella et al [19] for two reasons: One is that the sample size was insufficient to validate this secondary outcome, and the other is that the patients did not go through the nociceptive stimulation of surgery.

We believe that both groups had similar static and dynamic NRS scores at 24 h, as well as high NRS scores at 12-24 h, because the block resolved; in some studies, researchers have reported that analgesia lasts for 10-15 h after a perineural injection of long-acting local anesthetic [22–24], but this can’t meet the preoperative needs. The next study can consider adding adjuvant drugs [25, 26] or continuous nerve blocks to extend the duration of analgesia.

Previous studies believe that the nociceptive innervation of the hip joint mainly comes from the anterior wall of the hip capsule, including the femoral nerve, the obturator nerve, and the joint branch of the accessory obturator nerve [14]. The innervating nerves in the posterior wall of the hip capsule may be minimally involved in nociceptive innervation [27]. Current anatomy suggests that the mechanism of PENG block is that block of femoral nerve, obturator nerve, and joint branches of accessory obturator nerve exert analgesic effects [28]. Therefore, only the effect of PENG block on hip fracture pain was implemented in this study. Our results showed that PENG block significantly reduced resting and motor pain after hip fracture, but should not completely inhibit pain after hip fracture, suggesting that possibly other nerves, including the sciatic nerve may also be involved in the innervation of the hip. However, the role of the sciatic nerve in hip fracture pain, and whether the PENG block also covers the sciatic nerve, still need to be further studied.

The use of nerve block is an alternative for analgesia in patients with hip fractures when oral analgesics such as nonsteroidal anti-inflammatory drugs (NSAIDs) are contraindicated or predicted to be ineffective [29]. After patients with contraindications that may affect analgesia are excluded, early pain assessment and pain intervention are conducive to perioperative pain management and faster recovery [29]. Most studies on PENG block focus on the preoperative effect of the block rather than the postoperative effects, but our evaluation focused on both the preoperative and postoperative analgesia effects of PENG block. The few studies on the preoperative analgesia effect of PENG block are case reports and case series [13]. We provide a basis for the use of PENG block for early analgesia in elderly individuals with hip fractures.

This study has several limitations. First, this study had a relatively sample size and was not sufficiently powered to observe some rare complications. Second, some studies have shown that a pericapsular nerve block does not affect quadriceps muscle strength and can accelerate patient recovery [16, 20, 30]. Considering that the patient has a fracture, assessment of muscle strength may cause considerable harm to the patient, and we believe that it is unreasonable to evaluate the muscle strength of the patient before surgery. In addition, patients received intravenous flurbiprofen or the local block, and neither the subjects nor the staff were blinded. Finally, in the Group F, the patients were not injected with saline, and the patients were injected with saline, which we believe violates the principle of no harm and does not meet the ethical requirements.

In conclusion, compared to intravenous flurbiprofen, ultrasound-guided PENG block is a better early analgesic method for elderly individuals with hip fractures. Additional large-scale randomized clinical trials are needed.

### Supplementary Information

**Additional file 1: Table S1.** NRS scores at different times within the groups. **Table S2.** MAP, HR at different time points. **Table S3.** SpO_2_ at different time points.

## Data Availability

The datasets used and analyzed during the current study are available from the corresponding author on reasonable request.
