# Supplementary material for: Ultrasound-guided pericapsular nerve group (PENG) block for early analgesia in elderly patients with hip fractures: a single-center prospective randomized controlled study
Source: BMC Anesthesiol. 2023 Nov 23;23:383. doi: 10.1186/s12871-023-02336-1 (PMC10666449; doi:10.1186/s12871-023-02336-1)
Supplement: Supplementary file 1 — Additional file 1: Table S1. NRS scores at different times within the groups. Table S2. MAP, HR at different time points. Table S3. SpO2 at different time points. [file 12871_2023_2336_MOESM1_ESM.docx]

**Supplementary tables**

**Table S1** NRS scores at different times within the groups

|  |  | before intervention | 15 min after intervention | 30 min after intervention | 6 h after intervention | 12 h after intervention | 24 h after intervention |
| --- | --- | --- | --- | --- | --- | --- | --- |
| group F  (mean ±SD) ^a^ | Static NRS scores | 4.52±0.99 | 2.81±1.42 ^*^ | 2.48±1.43 ^*^ | 1.90±1.05 ^*^ | 2.74±1.36 ^*^ | 2.75±1.02 ^*^ |
|  | Dynamic NRS scores | 8.76±1.48 | 6.43±1.96 ^*^ | 6.00±1.93 ^*^ | 4.95±1.28 ^*^ | 4.29±1.26 ^*^ | 5.05±1.15 ^*^ |
| group P  (mean ±SD) ^a^ | Static NRS scores | 4.15±1.14 | 1.45±0.69 ^*^ | 0.75±0.72 ^*^ | 0.80±0.77 ^*^ | 1.13±0.74 ^*^ | 2.40±0.82 ^*^ |
|  | Dynamic NRS scores | 7.95±1.99 | 3.00±1.38 ^*^ | 2.30±1.22 ^*^ | 2.30±1.22 ^*^ | 2.55±1.05 ^*^ | 5.15±1.53 ^*^ |

Notes: Analyzed by Generalized Estimation Equations; ^a^ Marginal means from generalized estimation equations and SDs from raw data among persons with information at specific time points. * Shows a statistical difference when compared to before intervention (adjusted *P*<0.05).

**Table S2** MAP, HR at different time points

|  | F group (n=21) | P group (n=20) | *P* |
| --- | --- | --- | --- |
| MAP |  |  |  |
| before intervention | 81.43±7.788 | 85.70±9.707 | Interaction *P*=0.978  Time *P*=0.088  Group *P*=0.090 |
| 15 min after intervention | 80.10±7.395 | 84.15±7.393 |  |
| 30 min after intervention | 81.19±7.672 | 83.15±7.558 |  |
| HR |  |  |  |
| before intervention | 82.10±5.629 | 81.95±5.942 | Interaction *P*=0.068  Time *P*=0.844  Group *P*=0.084 |
| 15 min after intervention | 83.81±6.724 | 79.50±5.652 |  |
| 30 min after intervention | 83.86±6.421 | 80.50±4.685 |  |

Notes: Analyzed by repeated measures analysis of variance; Data are shown as mean ± SD; *P*<0.05 was considered statistically significant.

**Table S3** SpO_2_ at different time points

| Time | before intervention | 15 min after intervention | 30 min after intervention | *P* |
| --- | --- | --- | --- | --- |
| SpO_2_; (mean ± SD) ^a^ | 97.10±1.319 | 97.61±1.263 | 97.49±1.098 | <0.001 |

Notes: Analyzed by Generalized Estimation Equations; ^a^ Marginal means from generalized estimation equations and SDs from raw data among persons with information at specific time points. There was no interaction effect between time and group (*P*=0.071), SpO_2_ was not statistically significant in group (*P*=0.496); SpO_2_ was statistically significant in time (*P*<0.001). There was no statistically significant difference between 15 min after intervention and 30 min after intervention (*P*>0.05). SpO_2_ was slightly higher than before intervention at 15 min after intervention (*P*=0.000) and 30 min after intervention (*P*=0.025). *P*<0.05 was considered statistically significant.
